# Prevalence of Excessive Weight and Underweight and Its Associated Knowledge and Lifestyle Behaviors among Urban Private School-Going Adolescents in New Delhi

**DOI:** 10.3390/nu13093296

**Published:** 2021-09-21

**Authors:** Tina Rawal, Maartje Willeboordse, Monika Arora, Nitika Sharma, Gaurang P. Nazar, Nikhil Tandon, Constant P. van Schayck

**Affiliations:** 1 Health Promotion Division, Public Health Foundation of India, Gurgaon 122002, India; monika.arora@phfi.org; 2 Department of Family Medicine, Care and Public Health Research Institute, Maastricht University, P.O. Box 616, 6200 MD Maastricht, The Netherlands; maartje.willeboordse@maastrichtuniversity.nl (M.W.); onno.vanschayck@maastrichtuniversity.nl (C.P.v.S.); 3 Health Related Information Dissemination Amongst Youth (HRIDAY), Delhi 110029, India; nitikasharma519@gmail.com (N.S.); gaurang@hriday-shan.org (G.P.N.); 4 Department of Endocrinology and Metabolism, All India Institute of Medical Sciences, New Delhi 110029, India; nikhil_tandon@hotmail.com

**Keywords:** lifestyle behaviors, knowledge, body mass index, excessive weight, underweight, adolescents, India

## Abstract

With rapid urbanization and the Indian nutrition transition, Indian adolescents face a high risk of developing an energy imbalance. This study aims to assess the prevalence of excessive weight, underweight, and associated knowledge and lifestyle behaviors among private school-going adolescents in Delhi. A cross-sectional study was conducted in students (6th–7th grades) of eight randomly selected private schools in Delhi, India in 2019. A self-administered survey was used to assess students’ dietary-and-physical-activity-related knowledge and behavior. Anthropometric measurements (height, weight, and waist circumference) were also conducted. Out of 1567 participants, 7.2% were underweight, 61.3% normal, and 31.5% excess in weight. Underweight was associated with significantly more eating whilst studying for exams (relative risk ratio (RRR) 1.7 (1.0–2.9)). Excessive weight was associated with less incorrect knowledge on behaviors causing overweight (RRR 0.7 (0.5–0.9)), more often reading nutritional labels of packed food items (RRR 0.6 (0.4–0.9)), and less frequent vegetable-intake (RRR 0.7 (0.4–0.9)). Underweight students showed more suboptimal knowledge and unhealthy behaviors, whilst students with excessive weight showed more correct knowledge and healthy behaviors. This study highlights the immediate need for effective health-promoting interventions focused on the importance of healthy lifestyle at least in underweight adolescents.

## 1. Introduction

Globally, in both developed and developing nations, rates of overweight and obesity continue to rise [1]. Environmental factors and lifestyle choices including urbanization play major roles in the rising prevalence of obesity, are responsible for non-communicable diseases (NCDs) [2], and are largely a result of practices adopted during early ages. Energy imbalance resulting from the consumption of excess calories and inadequate physical activity is considered to be the major factor responsible for obesity [3]. Overweight and obese adolescents often grow into obese adults, who in turn are at higher risk of developing one or more NCDs [4]. Developing countries are experiencing an increase in overweight and obesity among all economic classes and all regions while being more prevalent in urban rather than rural areas [5,6]. In recent years, India has been facing a paradoxical co-occurrence of under- and over-nutrition as undernutrition continues to persist and causing both conditions to co-exist [7]. Regional studies conducted in India also highlighted the co-occurrence of over- and undernutrition among school-going adolescents [8,9]. A systematic review reported the combined prevalence of childhood overweight and obesity in India of 19.3%, which is a significant increase from the earlier prevalence of 16.3% reported in 2001–2005 [10]. Adolescents are exposed to an obesogenic environment, created by rapid urbanization and the nutrition transition (i.e., changes in dietary pattern due to increased accessibility, availability, and consumption of food high in fat, salt, and sugar) in India. Data from the Comprehensive National Nutrition Survey (CNNS, 2019) reported that every second child is affected by some form of over- or undernutrition and 1 in 10 adolescents have glycosylated HbA1c (between 5.7–6.4%) indicating high blood glucose values, which may be due to glucose disorders such as diabetes [11]. 

Several risk behaviors during the early years of life appear to be strongly correlated with both being underweight and excessive weight [12]. Both underweight and excessive weight students showed significantly vulnerable risk behaviors including substance use, mental health issues including depression, and violent behaviors. Suicide attempts were reported as critical risks for excessive students [13]. Therefore, it is imperative to monitor healthy living practices at an early age. Daily fruit/vegetable consumption and sufficient physical activity levels are good preventive factors for both underweight and excessive weight [14]. 

In India, there is a knowledge–practice gap among adolescents about eating and physical activity behaviors [15]. The lack of knowledge about healthy and unhealthy behaviors highlights the importance of carrying out regular surveillance for NCD risk factors and initiating educational programs for the prevention of NCDs amongst adolescents [16].

The primary aim of this article is to assess the prevalence of excessive weight and underweight and its associated knowledge and lifestyle behaviors among the urban private school-going adolescents (aged 11–12 years), in New Delhi. Secondly, we aim to study the correlates of BMI status (underweight, normal, excessive weight) with dietary and physical activity knowledge and behaviors among these participants.

## 2. Methodology

### 2.1. Study Design

This cross-sectional study was conducted in eight private schools of Delhi, India in 2019. Schools were randomly selected from the list of private schools governed by the Directorate of Education (DoE), Government of National Capital Territory (NCT) of Delhi. This study was limited to private schools in Delhi, as previous studies conducted in Delhi reported that the prevalence of excessive weight is significantly higher in private school students as compared to government school students [17]. 

### 2.2. Participants

Of the n = 1817 students enrolled in 6th and 7th grades (aged 11–12 years) in the eight participating schools, n = 1635 (89.9%) students participated in this study. As data on BMI were missing for n = 71 students, data from n = 1564 (95.8%) students were used for analysis. 

### 2.3. Measures

A self-administered survey was implemented in all the grades in these schools by a trained study team (convened under the guidance of the Principal Investigator) using a standardized protocol. The confidentially of responses was assured by using a unique ID not recognizable to the students. The survey was administered in English. The survey was adopted from other instruments that have been validated with adolescents and were extensively pilot-tested in India [6,17]. The survey assessed dietary knowledge; behavior including frequency of consumption of breakfast, fruits, vegetables, sugar-sweetened beverages, and energy-dense foods; food purchasing behavior; physical activity behavior including frequency of participation in 60 min per day of PA; use of available resources for physical activity; and duration of screen time. 

In addition to the survey, anthropometric measurements of students were conducted by a trained study team using a standardized protocol. Anthropometric measurements included height to the nearest 0.1 cm, weight to the nearest 100 g on an electronic scale, and waist circumference (WC). The WC was measured using a non-stretchable measuring tape, at a level midway between the lower rib margin and iliac crest with participants in standing position; the measurements were done to the nearest 0.1 cm. For all anthropometric data, the average of two readings for each student was used for analyses, using a protocol adapted to suit the Indian context [18]. For all anthropometric measurements, the students were asked to remove all excess clothing other than their regular school uniform, shoes, all items from pockets, watches, eyeglasses, belts, necklaces, and other jewelry. 

Students were grouped into five weight categories using the World Health Organization (WHO) age- and gender-specific body mass index (BMI) growth references [19]. These groups were underweight (thin BMI below −2 standard deviations (SD) and severe thin below −3 SD in the WHO reference population), normal (between −2 SD and 1 SD), overweight (between 1 SD and 2 SD), and obese (more than 2 SD). However, it was further categorized into three categories: normal; underweight (thin and severe thin); and excessive weight (overweight and obese). Students whose WC values were more than age- and gender-specific 70th percentile cut-off using Indian reference values were categorized as high WC [20]. 

### 2.4. Data Analysis

The descriptive analysis is presented using percentages and frequencies. Further, the potential correlates were assessed using exploratory data analysis. The dependent variable (BMI) was taken as a categorical variable with the categories underweight, normal, and excessive weight (based on World Health Organization (WHO) age- and gender-specific BMI growth references [19]). The socio-demographic variables, knowledge, dietary, and behavior factors were taken as independent variables. Related questions (dietary knowledge (3) and behavior (9); physical activity-related knowledge (1) and behavior (3)) are provided in the supplementary file (Supplementary Table S1) Cross tabulations, using Fisher’s exact/Chi-square were used to study bivariate associations between independent variables and BMI category. Where in the univariate analysis the associations were found to be significant, Tukey’s posthoc test was used to further study the differences between the specific BMI categories. Variables found to be significantly associated (*p* < 0.05) with the different categories of BMI (underweight, normal, and excessive weight) in the univariate analysis were included in the regression analysis as independent variables to assess the potential correlates with the BMI category. Since the dependent variable BMI had three categories, a multinomial regression analysis model was used to study the correlates of respective BMI categories (underweight and excessive weight), where normal weight was taken as a reference. All the estimates with *p*-values ≤ 0.05 were considered significant. All data analyses were conducted using STATA v.13 (StataCorp, LP, College Station, TX, USA).

## 3. Results

### 3.1. Socio-Demographic Characteristics 

Table 1 shows the descriptive statistics for socio-demographic variables and prevalence of under and excessive weight in the study sample. Overall, 1564 school-going adolescents participated in this study, of which 969 (61.9%) were boys and 595 (38.1%) were girls. In the sample, 7.2% of the students were underweight, 61.3% normal weight, and 31.5% were excess in weight. Around 17% of all adolescents had an unhealthy or increased WC as per their age. Socio-demographic variables were not different between participants of different weight categories, except for the father’s education, which was slightly lower for underweight students (*p* = 0.016) (see Supplementary file Table S2).

### 3.2. Associations of Knowledge of Diet and Physical Activity with BMI Status

**Figure 1:**
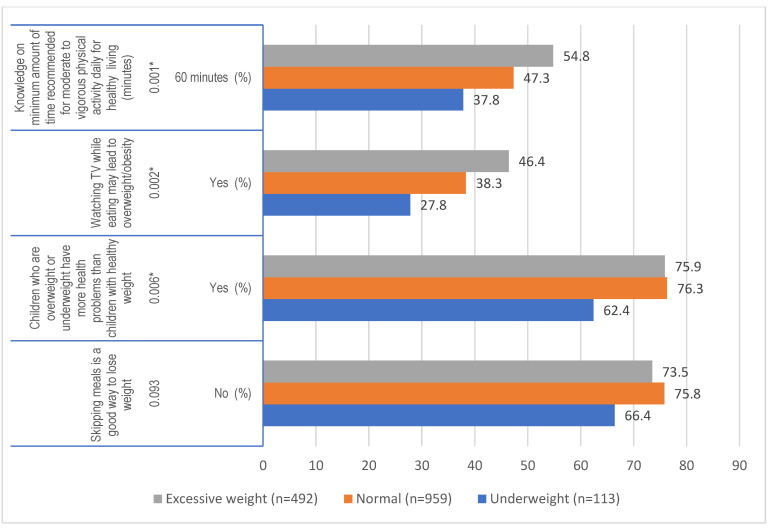
Univariate association of knowledge related to diet and physical activity and dietary behavior with BMI status of adolescence (* *p* values using Fisher’s Chi square *) * Correct responses are presented. Tukey’s post hoc test revealed that correct knowledge on the minimum amount of recommended moderate to vigorous physical activity was significantly lower in underweight vs. normal weight students (*p* = 0.003) and significantly higher in excessive weight vs. underweight students (*p* = 0.000); Correct knowledge on watching TV while eating may lead to overweight/obesity was significantly lower in normal vs. excessive weight students (*p* = 0.01) and underweight vs. excessive weight (*p* = 0.001); correct knowledge on health problems as a result of overweight was significantly lower for underweight vs. normal weight (*p* = 0.004) and underweight vs. excessive weight (0.009). See also Supplementary Table S2.

The percentage of students having the correct knowledge on healthy dietary and physical activity behaviors ranged between 27.8% and 76.3%. Three out of four knowledge variables differed significantly between groups (Figure 1). Participants with underweight had more often incorrect knowledge on diet and physical activity compared to normal weight students, as they had less knowledge on the recommended PA levels (37.8% vs. 47.3%, *p* = 0.003), and believed less often that ‘overweight or underweight students have more health problems’ (62.4% vs. 76.3% *p* = 0.004). Excessive weight students on the other hand, had more often correct knowledge on ‘watching TV while eating might lead to overweight’ (46.4% vs. 38.3%, *p* = 0.002). When comparing excessive weight with underweight students, excessive weight students had significantly more correct knowledge on three out of four knowledge variables. There were no significant associations in the physical activity behavior and BMI of the adolescents (*p* > 0.05).

Three out of nine dietary behaviors differed significantly between groups: daily vegetable intake (*p* = 0.015), eating more whilst studying for exams (*p* = 0.000), and studying nutrition labels on food packs (*p* = 0.002) (Table 2). Underweight participants reported eating more while studying for exams compared to normal-weight students (66.1 vs. 50.7%, *p* = 0.008). Excessive weight students showed healthier behaviors compared to normal weight students, as they less often reported they ate more while studying for exams (*p* = 0.05), and more often read nutritional labels of packaged food while purchasing them (*p* = 0.008).

### 3.3. Correlates of Excessive Weight and Underweight

In Table 3, further regression analyses are reported to identify the correlates of excessive weight and underweight. A total of 1198 observations could be included, as there were missing data in some of the independent variables. Most of the patterns observed in the univariate analyses were confirmed in the regression analyses. Students with mothers having senior secondary schooling were more likely to be underweight as compared to those with a professional degree (relative risk ratio (RRR) 2.6 (1.1–6.2)). Additionally, students with unhealthier eating behavior whilst studying for exams were more likely to be underweight as compared to those with healthier eating habits (RRR 1.7 (1.1–2.7)).

Students who had correct knowledge about the relation between watching TV while eating and overweight/obesity were less likely to be excessive in weight (RRR 0.7 (0.5–0.9)). Moreover, students with unhealthier eating behaviors whilst studying for exams were less likely to be excessive in weight as compared to those with healthier eating habits 0.8 (0.6–1.0). Students reading nutritional labels of packed food items were less likely to be excessive in weight (RRR for answer option rarely vs. most of the times 0.6 (0.4–0.9). Contrastingly, adolescents who consumed vegetables frequently (at least twice a day) were less likely to be excessive (RRR twice a day vs. once a day 0.7 (0.4–0.9)).

## 4. Discussion

The findings of this study show that 7.2% of the students were underweight, 61.3% normal weight, and 31.5% were excess in weight. The results highlighted that underweight students showed unhealthier dietary behaviors. Students who were eating more while studying for exams or having vegetable intake twice a day were less likely to be excessive in weight. However, there was no significant association between physical activity and weight of students. The percentage of students with excessive weight was alarmingly high, with 18.8% being overweight and 11.3% obese. The results of a study conducted in 2018 among Indian private school participants reported the percentage of overweight and obese students to be much lower with 13.2% overweight and 8.7% obese [6]. A systematic review conducted in 2016 reported the combined prevalence of overweight and obesity among children and adolescents in India as 19.3% [10]. In another recent study, the prevalence of overweight and obesity among school-going adolescents was found to be 9.9% and 14.0%, respectively [21]. The difference in the prevalence may be due to the regional difference between the states of India. However, it is most likely that currently, there is an increase in the prevalence of excess weight due to rapid economic and social transition which results in the nutrition transition, i.e., replacing traditional and healthier meals with fast food consumption including excess calories and food high in saturated and trans fats and sugar, and excessive use of technology leading to sedentary behavior. A systematic review also provides evidence for an overweight/obesity transition in school-aged children in Sub-Saharan Africa [22]. Another study conducted to understand the factors contributing to obesity in the early years in Latin America revealed that changes in socioeconomic conditions and urbanization have been the major contributors to the increasing prevalence in the region [23]. The COVID-19 crisis has led to even more unhealthy behaviors and a higher prevalence of overweight and obesity amongst adolescents, emphasizing the need to invest in effective interventions to prevent obesity starting at a young age [24]. Recently, a study conducted with adolescents during a lockdown period in Greece suggested prioritizing measures to increase physical activity, decrease sedentariness, and improve eating behavior for better well-being [25]. Findings of a systematic review emphasized the risk of elevated stress among this age group and the need to develop strategies to support families to cope with the current pandemic situation and ensure their children’s healthy development [26].

The present study emphasized that roughly 1/3 of participants have incorrect knowledge on very basic nutrition and physical activity-related behaviors, e.g., recommended physical activity guidelines. Other regional studies have also highlighted poor knowledge related to diet and exercise among school-going Indian adolescents [15,27]. In addition to the low knowledge levels, it was seen in all weight categories that dietary behaviors were mostly unhealthy. For example, more than half of all participants, irrespective of their BMI status, mentioned that they eat junk food multiple times a week. Findings of a study conducted with a sample of students from Central Michigan University also highlighted the need to improve physical activity, students’ knowledge of healthy and unhealthy diet habits, and nutritional knowledge [28].

Findings of this study reported that underweight students have slightly less knowledge on healthy behaviors and show slightly more behaviors that are unhealthy. Students with excessive weight, on the other hand, have the more correct knowledge on healthy behaviors, and also show healthier behaviors compared to normal weight students. This finding might be explained by either higher self-awareness of their weight and/or a family history of obesity in excessive weight students [29], resulting in a higher eagerness to learn about healthy behaviors and weight implications. Possibly, excessive weight students are more involved in dieting and/or exercise regimes, explaining their higher levels of knowledge and healthier behaviors. What is also a possibility is that due to weight stigmatization and body shaming, excessive weight students tend to answer the behavior questions with more socially desirable answers [30]. Body dissatisfaction among adolescents is also a major reason for unhealthy behaviors among both categories, excessive weight and underweight [31].

Our results corroborate and extend the findings from previous studies in India [32,33] and emphasize the need for immediate attention to curb the increasing prevalence of excessive weight among school-going adolescents. The increased availability, affordability, and accessibility to ready-to-eat food items including processed and energy-dense foods among adolescents living in urban areas result in almost all students showing high-risk factors of developing an imbalance in caloric intake. Decrease in physical activity and reduced energy expenditure along with television viewing and other sedentary behavior may also be significant factors that contribute to excessive weight in this age group [34]. Moreover, other studies show that the trend is more prevalent among families living in urban areas due to the easy availability and approachability of computer and mobile phone-based games and use the car or motor-bikes instead of walking or using a bicycle [30]. However, the findings of MyHeART (Malaysian Health and Adolescents longitudinal Research Team) study revealed that adolescents from rural areas are at higher risk of NCDs compared to their urban counterparts [35].

Risk factors for unhealthy behaviors including dietary and physical activity behavior are established in the early years of life [36]. Therefore, primordial prevention strategies must begin at a young age and continue into adolescence and preferably include all environments to which children are exposed. During adolescence, development and social changes take place which likely influence the dietary and physical activity patterns, including parent and peer influences, home and school food environments, and mass media [37]. As in adolescence, the immediate social group influence rises, schools might act as a facilitator and provide support by creating an enabling environment for healthy daily habits.

The strengths of this study include that schools were randomly selected, and anthropometrics were measures objectively. The sample size was big with over 1500 participants and the response rate was high with 89.9% of all eligible participants in schools participating in the study. Hence, the study findings are representative of urban school-going adolescents in private schools of Delhi and can be extrapolated to other urban areas in India. A limitation of this study is that it is restricted to one metropolitan city of India and responses to the questionnaire were self-reported. The findings may not be representative of rural areas, yet the large sample size makes the findings robust. Although knowledge is an important determinant for behavior change, sufficient knowledge alone is often not enough to change behavior in daily life. This is also reflected by our findings, as knowledge on healthy behaviors was highest amongst excessive weight students; however, this was not always reflected in actual healthy dietary behaviors. Several other important determinants of behavior change were not included in this study, such as attitudes, self-efficacy, and skills to adhere to behaviors following the social cognitive theory [38]. In future studies, we recommend including more determinants of health behavior following a well-established behavior change theory, as this provides more opportunities to design effective dietary and physical activity interventions [39].

## 5. Implications

Policymakers must recognize that children and adolescents who consume unhealthy diets and follow unfavorable lifestyle behaviors may have long-term health effects including unhealthy weight, which may lead to chronic conditions later in life. This study emphasizes that knowledge and lifestyle behaviors are suboptimal among adolescents in urban settings and leading to either being underweight or excess in weight. Knowledge and behaviors associated with diet and physical activity are vital for weight management. Therefore, there is a need for effective interventions to battle this unhealthy lifestyle epidemic, such as contextual comprehensive school and community-based targeted interventions. Policies focused on the availability, accessibility, and affordability of healthy food options in and around schools need to be enforced and monitored regularly. Moreover, to foster a supportive environment to improve dietary and physical activity knowledge and behavior, there is a need to limit advertising around unhealthy food products and promote easy to comprehend nutritional labeling for all to understand and practice healthy eating choices. Policies around fostering an enabling environment in and around school need to be created for the student of this age group to be motivated for being physically active. This study also highlights the need for larger studies to assess the prevalence of excessive weight and underweight among this age group regularly to revisit the kind of intervention needed in the future. At the same time, we should continue studying influential determinants and effective multi-factorial strategies for reducing overweight and obesity, to battle the steep increase in excessive weight amongst urban Indian adolescents.

## 6. Conclusions

The current study emphasized the co-occurrence of excessive weight and underweight amongst urban adolescents in India, and knowledge and behaviors on dietary and physical activity behaviors are often suboptimal in the entire population. Underweight students have slightly more often suboptimal knowledge and show unhealthier behaviors, whilst students with excessive weight have the more correct knowledge on healthy behaviors and also show healthier behaviors. Future studies should employ comprehensive theory-based interventions that target both underweight and excessive weight among adolescents and promote their overall well-being. Determining the prevalence and understanding the factors related to excessive weight and underweight among adolescents are vital for creating an enabling environment for them to foster healthy living practices. This study highlights the need for the stakeholders to reinforce the importance of a healthy lifestyle at home and school especially during the early years of life when lifestyle habits are being etched.

## Figures and Tables

**Table 1:** Socio-demographic variables of the study population.

Gender	*n* = 1564(%)
Boys	969 (61.9)
Girls	595 (38.1)
**Age in years (SD)**	12.5 (1.5)
Class 6	815 (52.1)
Class 7	749 (47.9)
**WC based on age**	
Increased WC in boys (*n* = 969)	165 (17.1)
Increased WC in girls (*n* = 595)	99 (16.6)
**Mean BMI (SD) in kg/m^2^**	18.9 (3.8)
**BMI category**	
Underweight (severe thin + thin) in *n* (%)	113 (7.2)
-Thin (*n*)	86
-Severe thin (*n*)	27
Normal in *n* (%)	959 (61.3)
Excessive weight (overweight + obese) in *n* (%)	492 (31.5)
-Overweight (*n*)	307
-Obese (*n*)	185

Abbreviations: SD—standard deviation, WC—waist circumference, BMI—body mass index.

**Table 2:** Univariate associations of dietary and physical activity behaviors with BMI status of adolescents.

Dietary Behaviours	Answer Options	Underweight (*n* = 113) N(%)	Normal (*n* = 959) N(%)	Excessive Weight (*n* = 492) N(%)	*p*-Value
Daily vegetable intake	Never (135)	14 (12.7)	80 (8.9)	41 (8.7)	0.015 *
	Once a day (273)	24 (21.8)	151 (16.9)	98 (20.9)	
	At least twice a day (434)	23 (20.9)	294 (32.8)	117 (24.9)	
	**At least thrice a day** (634)	49 (44.6)	371 (41.4)	214 (45.5)	
Fast/junk food (e.g., burgers, pizza, noodles etc.) intake frequency	**Never** (267)	15 (5.6)	162 (60.7)	90 (33.7)	0.142
	1–2 times/week (420)	29 (25.7)	271 (29.1)	120 (24.9)	
	3–6 times/week (627)	52 (46.0)	360 (38.6)	215 (44.6)	
	≥7 times/week (214)	17 (15.0)	140 (15.01)	57 (11.8)	
Eating breakfast	Never (81)	5 (4.5)	41 (4.4)	35 (7.3)	0.199
	Few times in a week (287)	22 (19.6)	173 (18.5)	92 (19.2)	
	**Every day** (1160)	85 (75.9)	723 (77.2)	352 (73.5)	
Eat more when out with friends	**No** (600)	36 (33.0)	362 (40.7)	202 (44.4)	0.082
	Yes (853)	73 (66.9)	527 (59.3)	253 (55.6)	
Eat more when out with family	**No** (271)	17 (16.0)	155 (17.3)	99 (21.3)	0.153
	Yes (1195)	89 (84.0)	741 (82.7)	365 (78.7)	
Eat more while studying for exams	**No** (724)	36 (33.9)	433 (49.3)	255 (56.0)	0.000 *
	Yes (715)	70 (66.1)	445 (50.7)	200 (44.0)	
Eat more when physically active	**No** (578)	35 (35.4)	343 (39.1)	200 (45.2)	0.052
	Yes (841)	64 (64.7)	535 (60.9)	242 (54.8)	
Eat more when angry	**No** (946)	63 (61.8)	566 (64.4)	317 (70.3)	0.063
	Yes (486)	39 (38.2)	313 (35.6)	134 (29.7)	
Reading nutritional labels on food packs before purchasing them	Never (197)	17 (15.3)	127 (13.7)	53 (11.1)	0.002 *
	Rarely (269)	17 (15.3)	179 (19.2)	73 (15.1)	
	Sometimes (617)	58 (52.3)	371 (38.9)	188 (39.3)	
	**Most of times** (437)	19 (17.1)	253 (27.2)	165 (34.5)	
Apart from eating activities done during lunch break	Sat down and chat with friends (508)	35 (35.4)	302 (33.6)	156 (34.1)	0.28
	Stood or walked around (283)	16 (16.2)	162 (18.0)	93 (20.3)	
	Played active games (595)	37 (37.4)	360 (40.0)	173 (37.8)	
	Completed homework (95)	11 (11.0)	49 (5.5)	25 (5.5)	
	Others (40)	0	26 (2.9)	11 (2.4)	
Number of days in a week in which you were involved in any form of physical activity	Every day (61)	2 (1.9)	44 (4.8)	15 (3.2)	0.35
	1–2 days (533)	29 (28.7)	333 (36.4)	171 (36.9)	
	3–4 days (234)	19 (18.8)	145 (15.8)	70 (15.1)	
	5–6 days (400)	27 (26.8)	241 (26.3)	132 (28.5)	
	Never (252)	24 (23.8)	153 (16.7)	75 (16.3)	
Duration of vigorous physical activity on a typical day	30 min–1 h (1123)	75 (70.1)	662 (72.9)	339 (72.3)	0.37
	2–3 h (235)	15 (14.0)	138 (15.2)	73 (15.6)	
	≥4 h (44)	1 (0.9)	22 (2.4)	17 (3.6)	
	Never (147)	16 (15.0)	86 (9.5)	40 (8.5)	

Preferred behaviors are expressed in bold. * Tukey’s HSD test revealed that the average daily vegetable intake did not differ significantly among any of the three weight categories; reading nutritional labels of packaged food while purchasing was significantly more frequently done in excessive weight vs. normal (*p* = 0.01) and excessive weight vs. underweight (*p* = 0.03); eating more whilst studying during exams was significantly lowest in excessive weight students (excessive vs. normal weight (*p* = 0.05), excessive vs. underweight students (*p* = 0.00), and highest amongst underweight students (underweight vs. normal weight (*p* = 0.008). See also Supplementary Table S3.

**Table 3:** Correlates of excessive weight and underweight (relative to normal weight) for dietary and PA knowledge and dietary behaviors.

Base Outcome	Underweight RRR, (95% CI)	Excessive Weight RRR, (95% CI)
**Education of mother**		
Advanced professional degree (e.g., post-graduation, Ph.D., etc.)	Ref	Ref
Graduate	1.3 (0.6–2.9)	0.8 (0.6–1.2)
Up to senior secondary	**2.6 (1.1–6.2)**	0.9 (0.6–1.4)
Up to middle school	2.0 (0.7–5.6)	1.2 (0.7–2.0)
No formal schooling	1.8 (0.5–6.5)	0.7 (0.3–1.6)
**Education of father**		
Advanced professional degree (e.g., post-graduation, Ph.D., etc.)	Ref	Ref
Graduate	1.1 (0.6–1.9)	1.2 (0.9–1.7)
Up to senior secondary	0.7 (0.3–1.4)	0.6 (0.4–1.0)
Up to middle school	1.0 (0.4–2.4)	0.8 (0.5–1.4)
No formal schooling	0.3 (0.0–3.3)	0.8 (0.3–2.7)
**Knowledge: Children who are overweight or underweight have more health problems than children with a healthy weight**		
-Yes	Ref	Ref
-No	1.6 (0.9–2.6)	1.06 (0.8–1.5)
**Knowledge: Watching TV while eating may lead to overweight/obesity**		
-Yes	Ref	Ref
-No	1.5 (0.9–2.5)	**0.7 (0.5–0.9)**
**Knowledge: Minimum amount of time recommended for moderate to vigorous physical activity daily for healthy living (minutes)**		
<60 min	Ref	Ref
>60 min	0.7 (0.4–1.1)	1.3 (0.9–1.6)
**Behavior: Read the nutritional labels of packed food items while purchasing them**		
Most of times	Ref	Ref
Sometimes	1.7 (0.9–3.2)	0.9 (0.6–1.2)
Rarely	0.9 (0.5–2.1)	**0.6 (0.4–0.9)**
Never	1.5 (0.7–3.1)	0.7 (0.4–1.1)
**Behaviour: Daily vegetable intake frequency**		
Once a day	Ref	Ref
At least twice a day	0.6 (0.3–1.2)	**0.7 (0.4–0.9)**
At least thrice a day	0.8 (0.4–1.5)	0.9 (0.7–1.3)
Never	1.0 (0.4–2.2)	0.9 (0.5–1.5)
**Behaviour: Eat more while studying for exams**		
No	Ref	Ref
Yes	**1.7 (1.1–2.7)**	**0.8 (0.6–1.0)**

RRR: Relative risk ratio. Estimates derived using multinomial logistic regression taking normal weight as the reference category, the total included observations = 1163. *p* values less than 0.05 in the univariate associations were included in the regression analysis models. The bold estimates mean statistically significant differences compared to normal weight *p* < 0.05.

## Data Availability

The data presented in this study are available on request from the corresponding author.

## Supplementary Materials

The following are available online at https://www.mdpi.com/article/10.3390/nu13093296/s1 Table S1: Description of independent variables, Table S2: Differences in socio-demographic factors between overweight/obese adolescents and normal/underweight adolescents, Table S3: Tukey Post hoc tests for group differences in behavior and knowledge variables
