# Supplementary material for: Prevalence of Excessive Weight and Underweight and Its Associated Knowledge and Lifestyle Behaviors among Urban Private School-Going Adolescents in New Delhi
Source: Nutrients. 2021 Sep 21;13(9):3296. doi: 10.3390/nu13093296 (PMC8472350; doi:10.3390/nu13093296)
Supplement: Supplementary file 1 [file nutrients-13-03296-s001.zip › nutrients-1354923-supplementary.pdf]

Supplementary Files

Supplementary Table S1: Description of independent variables

Dietary knowledge	Q1 How much do you agree that watching TV while eating may lead to overweight/obesity?<br>Q2 How much do you agree that children who are overweight or underweight have more health problems than children with healthy weight?<br>Q3 How much do you agree that skipping meals is a good way to lose weight?
Physical activity knowledge	Q4 The minimum amount of time recommended for moderate to vigorous physical activity daily (running, cycling, brisk walking, jogging, play sports etc.) for healthy living among children and youth is:<br>- Less than 60 minutes<br>- 60 minutes
Dietary Behaviour	Q5 How often do you read the nutritional labels of packed food items while purchasing them?<br>Q6 Do you eat more than usual when you are studying for exams?<br>Q7 Do you eat more than usual when you are physically active?<br>Q8 How many times do you eat Vegetables (katoris/bowl)/day?<br>Q9 In a typical week, on how many days do you eat breakfast?<br>Q10 In a typical week, how often do you eat fast/ junk food (e.g. Burger, Pizza, noodles, etc.)?<br>Q11 Do you eat more than usual when you are out with friend/s?<br>Q12 Do you eat more than usual when you are out with the family?<br>Q13 Do you eat more than usual when you are angry?
Physical activity behaviour	Q14 Over the last 5 school days, what did you do most of the time during lunch break (apart from eating)?<br>Q15 In a typical week, on how many days you are involved in extracurricular activities at school which require being physically active (e.g. dancing, playing games, gymnastics, etc.)?<br>Q16 On a typical day, how many hours do you spend doing vigorous physical activity (e.g: running, fast cycling, fast swimming, or moving heavy loads)

Supplementary Table S2: Differences in socio-demographic factors between overweight/obese adolescents and normal/underweight adolescents.

BMI Categories	Underweight (n=113) N(%)	Normal (n=959) N(%)	Excessive weight (n=492) N(%)	p-value
**Gender**				0.428
Boys (969)	72(63.7)	582(60.7)	315(64.0)	
Girls (595)	41(36.3)	377(39.3)	177(35.9)	
**Class**				0.425
6 (815)	53(46.9)	498(51.9)	264(53.7)	
7 (749)	60(53.1)	461(48.1)	228(46.3)	
**Education of father**				**0.016***
Advanced professional degree (e. g. PG, PhD etc.) (389)	21(19.6)	238(26.2)	130(28.6)	
Graduate (552)	42(39.3)	322(35.3)	188(41.4)	
Up to Senior Secondary (316)	26(24.3)	218(24.0)	72(15.9)	
Up to Middle school (183)	17(15.9)	108(11.9)	58(12.8)	
No formal schooling (28)	1(0.9)	21(2.6)	6(1.3)	
**Education of mother**				0.051
Advanced professional degree (e. g. PG, PhD etc.) (263)	11(10.1)	163(17.6)	89(19.7)	
Graduate (569)	33(30.3)	356(38.5)	180(39.6)	
Upto Senior Secondary (362)	37(33.9)	228(24.7)	97(21.3)	
Upto Middle school (231)	23(21.1)	136(14.7)	72(15.8)	
No formal schooling (64)	5(4.6)	42(4.5)	17(3.7)	
**Father's occupation**				0.155
Professional (539)	28(5.2)	344(63.9)	167(30.9)	
Semi-professional (242)	16(6.6)	147(60.7)	79(32.7)	

Clerical, shop owner, farmer (222)	18(8.1)	129(58.1)	75(33.8)	
Worker (296)	21(7.1)	187(63.2)	88(29.7)	
Unemployed (40)	7(17.5)	23(57.5)	10(25)	
**Mother's occupation**				0.260
Professional (189)	7(7.2)	127(15.5)	55(13.7)	
Semi-professional (67)	6(6.2)	37(4.5)	24(5.9)	
Clerical, shop owner, farmer (45)	3(3.1)	23(2.8))	19(4.7)	
Worker (87)	5(5.2)	54(6.6)	28(6.9)	
Unemployed (931)	76(78.4)	579(70.6)	276(68.8)	

Supplementary Table S3: Tukey Post hoc tests for group differences in behavior and knowledge variables

**Variables**	**Normal versus Underweight**	**p-value**	**Normal versus Excessive weight**	**p-value**	**Underweight versus Excessive weight**	**p-value**
	**(Mean difference)**		**(Mean difference)**		**(Mean difference)**	
**Behaviour: Daily vegetable intake**	0.06	0.79	0.003	0.79	0.06	0.99
**Behaviour: Eat more while studying during exams**	0.15	**0.008**	0.07	**0.05**	0.22	**0.000**
**Behaviour: Read nutritional labels of packaged food while purchasing**	0.09	0.59	0.16	**0.008**	0.26	**0.03**
**Knowledge: minimum amount of recommended moderate to vigorous physical activity**	0.34	**0.003**	0.12	0.085	0.47	**0.000**
**Knowledge: watching TV while eating may lead to overweight/obesity**	0.15	0.086	0.08	**0.011**	0.19	**0.001**
**Knowledge: children who are overweight or underweight have more health problems than children with a healthy weight**	0.14	**0.004**	0.00	0.989	0.13	**0.009**
